# *ATM* Serine/Threonine Kinase and its Role in Pancreatic Risk

**DOI:** 10.3390/genes11010108

**Published:** 2020-01-17

**Authors:** Neha Nanda, Nicholas J. Roberts

**Affiliations:** 1 Department of Pathology, Department of Pathology, The Johns Hopkins University School of Medicine, Baltimore, MD 21287 USA; nnanda3@jhmi.edu; 2 The Sol Goldman Pancreatic Cancer Research Center, The Johns Hopkins University School of Medicine, Baltimore, MD 21287, USA; 3 Department of Oncology, The Johns Hopkins University School of Medicine, Baltimore, MD 21287, USA

**Keywords:** *ATM*, pancreatic ductal adenocarcinoma, pancreatic cancer, genetics, predisposition

## Abstract

Next-generation sequencing has led to the recent discovery of several novel pancreatic cancer susceptibility genes. These genes include ataxia telangiectasia mutated (*ATM*), a serine/threonine kinase that is an integral component of DNA repair. Pathogenic germline *ATM* variants are frequently identified in patients with pancreatic ductal adenocarcinoma (PDAC) with and without a family history of the disease. Loss of *ATM* is also a frequent somatic event in the development of PDAC. These discoveries have advanced our understanding of the genetic basis of pancreatic cancer risk and will impact patient care through appropriate patient–risk stratification; personalized screening and early detection efforts; and, for some, targeted therapy.

## 1. Introduction

Pancreatic ductal adenocarcinoma (PDAC) is the third-leading cause of cancer-related deaths in the U.S., with a five-year survival rate of only 9% [1]. Over 50,000 new cases of PDAC are diagnosed each year in the U.S., and more than 40,000 die due to this disease [2]. By 2030, PDAC is estimated to become the second-leading cause of cancer-related deaths in the United States. [1].

Approximately 10% of patients with PDAC have a first-degree relative with the disease and are termed familial pancreatic cancers (FPC) [3]. Aggregation of PDAC in these families can be attributed to genetic, environmental, and stochastic factors [4]. Inherited genetic factors are thought to underlie susceptibility in up to half of FPC families [5,6,7], with pathogenic germline variants in pancreatic cancer susceptibility genes, including *ATM*, *BRCA1*, *BRCA2*, *CDKN2A*, *CPA1*, *CPB1*, *MLH1*, *MSH2*, *MSH6*, *PALB2*, *PMS2*, *PRSS1*, and *STK11*, associated with a high risk of developing PDAC [8]. Knowledge of the genes responsible for increased risk in these families may be beneficial for appropriate risk-based screening programs for pancreatic and extra-pancreatic malignancies in family members carrying a pathogenic germline variant of a pancreatic cancer susceptibility gene. When instigated, screening programs may detect cancers early when treatment outcomes are improved [9]. Furthermore, in patients with PDAC, pathogenic germline variants may hint at underlying biological susceptibilities that can be exploited therapeutically, as is the case with poly (*ADP*-ribose) polymerase-1 (PARP-1) inhibition in *BRCA2*-deficient tumors and immunotherapy in mismatch repair-deficient tumors [10]. 

**Figure 1:**
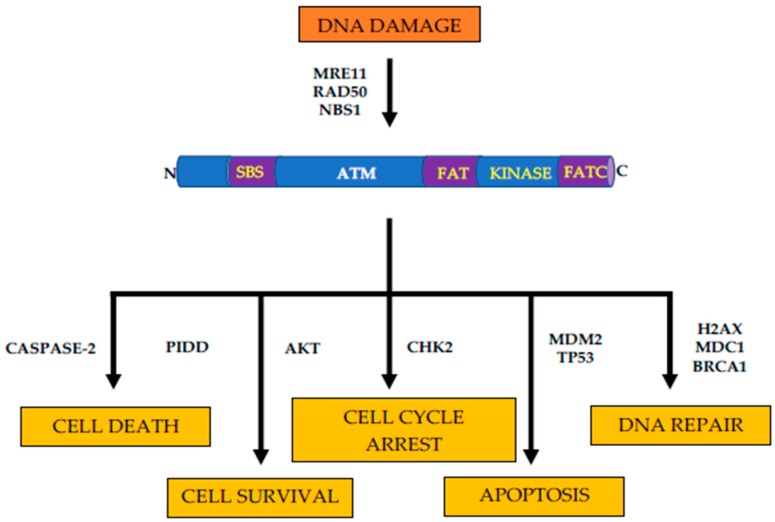
Structure and functions of ATM kinase. Schematic representation of ATM structure and cellular responses to DNA damage. N = n-terminus, C = c-terminus, SBS = substrate binding site, FAT = FAT domain, KINASE = kinase domain, and FATC = FATC domain. DNA damage induces autophosphorylation via MRN. Cellular responses to ATM activation include DNA repair, apoptosis, cell cycle arrest, cell survival, and cell death mediated through various downstream targets.

*ATM* serine/threonine kinase (*ATM*) is a member of the phosphoinositide 3-kinase-related protein kinase (*PIKK*) family and is an integral component of DNA damage response [11]. In response to DNA double-strand breaks, *ATM* phosphorylates and activates a network of sensor proteins, downstream kinases, and their substrates, resulting in activation cell cycle checkpoints, cell cycle arrests, and apoptosis [12]. Similar to other members of the *PIKK* family, such as *ATR*, *DNAPKcs*, *mTOR,* and *SMG1*, *ATM* shares domain structure and organization. Specifically, the kinase domain of *ATM* is flanked by c-terminal conserved *FAT* (*FRAP*, *ATR*, and *TRRAP* proteins); *PIKK* kinase; and *FATC* domains [13]. These domains regulate the kinase activity of *ATM* through the binding of regulatory proteins and subsequent post-translational modifications (Figure 1) [14].

*ATM* is also the cause of ataxia-telangiectasia (AT), a rare autosomal recessive disorder characterized by neurodegeneration, radiation hypersensitivity, immunodeficiency, and cancer predisposition [15,16]. Heterozygous carriers of pathogenic germline *ATM* variants have an increased risk of several cancer types, including hematopoietic, breast, pancreatic, and gastric cancer [17,18]. Identifying individuals with a pathogenic germline *ATM* variant, and therefore, an increased risk of cancer, is critical to early detection efforts that hope to improve patient care by detecting PDAC before it has spread to other sites in the body. 

In this review, we discuss the role of *ATM* in susceptibility to PDAC, as well as screening and early diagnosis of PDAC in heterozygous carriers of pathogenic germline variants in pancreatic cancer susceptibility genes, such as *ATM*. 

## 2. Pathogenic Germline *ATM* Variants in Patients with Familial Pancreatic Cancer

Next-generation sequencing of familial pancreatic cancer (FPC) patients provided the first conclusive evidence that *ATM* was a pancreatic cancer susceptibility gene [19]. In this study, Roberts and colleagues conducted whole-genome sequencing of 16 patients with FPC from six families and whole-exome sequencing of 22 patients with FPC from 10 families. The authors employed a filter-based approach to putatively pathogenic germline-coding variants and identified two families where all sequenced-affected members carried nonsense germline *ATM* variants that were rare in population-based variant databases (<0.005 minor allele frequency). Moreover, in one patient with available pancreatic tumor tissue, loss-of-heterozygosity (LOH) at the *ATM* locus was demonstrated with retention of the nonsense variant, demonstrating that *ATM* conformed to the classic two-hit model for tumor suppressor genes [20]. To verify the association between pathogenic germline *ATM* variants and PDAC, the authors sequenced the entire coding region of *ATM* in 166 FPC patients and 190 healthy spouse controls and identified pathogenic germline *ATM* variants in four patients (2.4%), compared to zero controls (0%). This association was stronger in those families with three or more affected family members, where four out of 87 patients with FPC (4.6%) carried a pathogenic germline *ATM* variant. 

Several subsequent studies have provided additional evidence to support the role of *ATM* as a pancreatic cancer susceptibility gene. Grant and colleagues analyzed the prevalence of pathogenic germline variants in pancreatic cancer patients using a multiple-gene panel of established pancreatic cancer susceptibility genes. In this study, 11 out of 290 patients with PDAC had a pathogenic germline variant in a pancreatic cancer susceptibility gene, including three in *ATM*, one of which had a family history of FPC [21]. Takai and colleagues determined the prevalence of pathogenic germline variants in pancreatic cancer susceptibility genes in 54 patients with FPC and identified two patients with pathogenic germline *ATM* variants, indicating that *ATM* is also a frequent underlying cause of pancreatic cancer in Japanese patients [22].

Recent large-scale sequencing studies of pancreatic cancer patients have shown that pathogenic germline *ATM* variants are one of the most frequently identified germline alterations in pancreatic cancer patients. In a study by Hu and colleagues, multigene panel testing of 96 patients with PDAC found pathogenic germline *ATM* variants in four individuals, representing 31% of all pathogenic germline variants identified (four out of 14) [23]. Interestingly, one patient with pathogenic germline *ATM* variant had a family history of FPC. Roberts and colleagues conducted whole-genome sequencing of 638 patients with FPC and identified pathogenic germline *ATM* variants in 19 families (3.4%) [24]. In their study, Roberts and colleagues also noted non-segregation of pathogenic germline variants found in pancreatic cancer susceptibility genes with PDAC in several kindreds. This finding should be considered when designing studies to identify novel susceptibility genes. Similarly, Chaffee and colleagues found pathogenic germline *ATM* variants in six out of 185 patients with FPC using a multigene panel test [8]. In one of the largest studies to date of 3030 pancreatic cancer patients, pathogenic germline *ATM* variants were identified in 69 patients (2.3%; 95% confidence interval (CI), 4.38–7.33) [25], including 11 patients with FPC. A similar study by Hu and colleagues used multigene panel testing and identified pathogenic germline *ATM* variants in 18 out of 475 patients with PDAC (3.8%) [26]. Finally, in a multicenter study of patients with PDAC, pathogenic germline *ATM* variants were found in four out of 289 patients (1.4%) using a next-generation sequencing custom gene panel. Of note, two of the patients harboring pathogenic germline *ATM* variants had family histories of pancreatic cancer [27]. Together, these studies suggest that pathogenic germline *ATM* variants are a frequent cause of FPC, being found in up to 3.8% of patients with a family history of the disease.

Interestingly, some patients with FPC have pathogenic (or rare missense) germline variants in multiple pancreatic cancer susceptibility genes [24,28]. However, the effects of such observations on the risk of pancreatic cancer and pancreatic tumorigenesis is unknown. 

## 3. Pathogenic Germline *ATM* Variants in Patients without a Family History of Pancreatic Cancer

Ninety percent of patients with PDAC do not have a family history of the disease that would meet the criteria for FPC. Until recently, the role of pathogenic germline variants in these patients without a family history of PDAC was poorly understood. Several recent studies have assessed the prevalence of pathogenic germline variants in pancreatic cancer susceptibility genes and other hereditary cancer predisposition genes in patients with PDAC without or unselected for a family history and found that between 1.0–4.2% of patients have a pathogenic germline *ATM* variant (Table 1). Shindo and colleagues sequenced 32 pancreatic cancer susceptibility genes, hereditary cancer predisposition genes, and candidate pancreatic cancer susceptibility genes in a series of 854 patients with PDAC [3]. They identified pathogenic germline *ATM* variants in 10 patients (1.2%), indicating that *ATM* plays a previously unappreciated role in susceptibility to pancreatic cancer even in patients without a family history of the disease. Further studies have confirmed the prevalence of pathogenic germline variants in pancreatic cancer susceptibility genes and hereditary cancer predisposition genes in patients with PDAC and other cancer types. A study by Yirgelun and colleagues identified four pathogenic germline *ATM* variants in 289 patients with PDAC, including two without a family history of the disease [27]. In another study by Mandeleker and colleagues using a 410-gene sequencing panel, five pathogenic germline *ATM* variants were identified in 176 patients with PDAC [29]. The cancer genome atlas (TCGA) network has performed deep whole-exome sequencing on PDAC specimens where pathogenic germline *ATM* variants were observed in three out of 149 (2%) patients with PDAC, along with pathogenic germline variants in other established pancreatic cancer predisposition genes in eight patients [30]. In a prospective multicenter study to characterize pathogenic germline variants in pancreatic cancer susceptibility genes in 298 unselected patients with PDAC by Brand and colleagues, pathogenic germline *ATM* variants were observed in 10 patients (3.3%) in the most frequently encountered gene [31]. Similarly, a study by Smith and colleagues using gene sequencing assessed *ATM* in two series of patients: a French-Canadian series of 114 patients with PDAC and a Quebec pancreas cancer study series of 236 patients with PDAC. In this study, one (0.9%) pathogenic germline *ATM* variant in the French-Canadian series and three (1.3%) pathogenic germline *ATM* variants in the Quebec pancreas cancer study series were identified, consistent with the findings of other studies [32]. Furthermore, a study using a multiple-gene testing panel identified pathogenic germline *ATM* variants in 11 out of 615 unselected patients with exocrine pancreatic malignancy, the majority of which had a diagnosis of PDAC. Moreover, analysis of somatic *ATM* alterations identified *ATM* LOH in 62.5% of patients with a pathogenic germline *ATM* variant, compared to 25% of patients without a pathogenic germline *ATM* variant, eluding to an important role of *ATM* in pancreatic tumorigenesis [33]. Finally, Singhi and colleagues performed targeted genomic profiling in 3594 tumor samples from unselected patients with PDAC and found pathogenic germline *ATM* variants in 48 patients (1.3%). Interestingly, the number of pathogenic germline *ATM* variants identified in male patients was greater than the number in female patients, and this difference was statistically significant [34].

Importantly, given the frequency of pathogenic germline variants in pancreatic cancer susceptibility genes in patients without a family history of PDAC and the clinical utility of such findings, the national comprehensive cancer network (NCCN) clinical guidelines changed to recommend the consideration of germline genetic testing in all patients with PDAC [35]. Of note, a recent analysis by Mandelker and colleagues used tumor-only sequencing data to detect germline alterations in cancer susceptibility genes. This may provide a pathway to increased germline testing in patients with pancreatic cancer. Specifically, a robust filtering of genomic calls, including variant allele frequency, was used to overcome the inherent difficulty in differentiating somatic and germline calls. The reduced number of germline variants to follow-up due to the improved specificity of germline calls may increase future germline analyses in tumor-only clinical sequencing data [36].

## 4. Pathogenic Germline *ATM* Variants in Patients with PanIN and IPMN

PDAC develops from noninvasive precursor lesions, such as pancreatic intraepithelial neoplasia (PanIN) and intraductal papillary mucinous neoplasms (IPMNs). PanINs are microscopic lesions that arise in the context of inflammation and transform into invasive PDAC [37]. Murphy and colleagues analyzed laser–capture microdissected PDACs and adjacent PanINs by exome sequencing and found *ATM* somatically mutated in two tumors, one with an adjacent PanIN harboring the same *ATM* mutation. *ATM* somatic mutations, however, were not found in PanINs without adjacent tumors, indicating somatic mutations of *ATM* may be linked to tumor progression [38]. Similarly, an in vivo study of AKC mice (p48^Cre/+^, Kras^G12D/+^, and Atm^−/−^) demonstrated an increased number of low and high-grade PanINs, compared to KC mice (p48^Cre/+^, Kras^G12D/+^, and Atm^+/+)^. These observations suggest that loss of *ATM* may be involved in early stages of PDAC tumorigenesis [39].

Intraductal papillary mucinous neoplasms (IPMNs) are macroscopic pancreatic cancer precursor lesions that are commonly diagnosed in the population, being present in up to 2.6% of patients undergoing contrasted–enhanced multidetector computed tomography for nonpancreatic disease [40]. As IPMNs are macroscopic and identifiable by noninvasive imaging techniques, they represent a potential target for the early detection of PDAC. However, as IPMNs are common in the population, characteristics to stratify patients for risk-appropriate screening protocols, such as inherited risk, are needed. 

In a recent retrospective study, Skaro and colleagues sequenced 315 unselected patients with surgically resected IPMNs using a multigene. They found that nearly 3% of patients with surgically resected IPMNs had a pathogenic germline variant in a pancreatic cancer susceptibility gene. Furthermore, pathogenic germline *ATM* variants were identified in five patients (1.6%) [41]. Interestingly, those patients with a pathogenic germline variant in a pancreatic cancer susceptibility gene were more likely to have microscopic invasive carcinoma. Further studies are necessary to determine the prevalence of pathogenic germline *ATM* variants in patients with IPMN that have not had surgical resections and the magnitude of the risk of progression to PDAC associated with pathogenic germline *ATM* variants in patients with IPMN.

## 5. Somatic *ATM* Alterations in PDAC

Initial evidence suggesting a role for somatic *ATM* alterations in pancreatic tumorigenesis came from genomic studies of patients with FPC. Roberts and colleagues identified somatic LOH of the *ATM* locus in the tumor of a patient with a pathogenic germline *ATM* variant. However, Grant and colleagues identified similar somatic LOH events encompassing the *ATM* locus in patients with PDAC and a pathogenic germline *ATM* variant; the lost allele was the pathogenic variant [42]. TCGA network has observed similar retention of a wild-type *ATM* allele in the tumor of a patient with a pathogenic germline *ATM* variant [30].

In the face of these conflicting studies in patients with pathogenic germline *ATM* variants, several studies have comprehensively assessed somatic alterations that occur in PDAC [30,34,43,44]. Biankin and colleagues provided evidence of frequent somatic *ATM* alterations in PDAC when they analyzed 99 pancreatic ductal adenocarcinomas without any family history using whole-exome sequencing. They found that 8% of PDAC tumor samples had somatic *ATM* alterations, either mutations, LOH, or both [45]. Subsequently, somatic *ATM* alterations were identified in 2–18% of PDACs in whole-genome, whole-exome, or targeted-sequencing studies [30,38,46,47,48,49,50]. The observed frequency of somatic *ATM* alterations in PDAC is supported by large immunohistochemical studies of surgically resected PDAC samples. Kim and colleagues assessed the expression of *ATM* in 396 patients with PDAC and observed a loss of *ATM* expression in more than 50 patient samples (12.6%), with more frequent loss of expression in patients with FPC compared to patients without a family history of PDAC (24.5% versus 11%). Interestingly, loss of *ATM* expression was associated with overall survival rates in a *TP53*-dependent manner. That is, patients with loss of *ATM* expression and normal *TP53* expression had decreased overall survival compared to patients with loss of *ATM* expression and abnormal *TP53* expression (HR = 2.63; 95% CI, 1.22–5.67), indicating that *ATM* expression may be an important prognostic factor for survival in patients with PDAC [51].

in vivo studies have also provided evidence to elucidate the role of *ATM* in pancreatic tumorigenesis. In a study by Russell and colleagues, *Atm* deficiency resulted in increased pancreatic lesions with oncogenic *KRAS*, deregulated TGF-β signaling, and epithelial-to-mesenchymal transition [39]. Similarly, partial or total *Atm* loss resulted in accumulated DNA damage and progression of pancreatic lesions [52].

Somatic *ATM* mutations have been identified in pancreatic cancers other than PDAC. Jiao and colleagues whole-genome sequenced 23 cases of acinar cell carcinoma, a tumor type associated with a poor prognosis that accounts for approximately 2% of pancreatic cancer cases, and found one somatic *ATM* mutation (4%) [53]. Furthermore, Corbo and colleagues sequenced 16 ampula of Vater cancers using a multigene panel targeting kinases and identified one sample with a somatic *ATM* mutation [54]. These data indicate that *ATM* may be important in the development of pancreatic cancers beyond its role in the development of PDAC. 

## 6. Diagnostic and Therapeutic Implications

Early detection of pancreatic cancers is associated with improved five-year survival rates [55]. Identifying individuals that would benefit from clinical surveillance is critical to early detection efforts. Family history and the inheritance of pathogenic germline variants in pancreatic cancer susceptibility genes are associated with a significantly increased risk of PDAC, and therefore, are factors to consider when stratifying patients for risk-based clinical surveillance. 

While studies of clinical surveillance for PDAC in patients with pathogenic germline *ATM* variants are lacking, the benefit of clinical surveillance in this patient population can be gleamed from studies of patients with FPC and patients with pathogenic germline variants in another pancreatic cancer susceptibility gene. In a study of 214 patients with a family history of PDAC; 178 patients with pathogenic germline *CDKN2A* variants; and 19 patients with pathogenic germline *BRCA1*, *BRCA2*, or *PALB2* variants, a clear benefit of clinical surveillance in patients with a pathogenic germline *CDKN2A* variant was found. Specifically, PDAC was detected in 13 patients (7.3%) and the majority underwent resection. The five-year survival rate for these patients was 24%, highlighting the benefits of early detection and surgical intervention [56]. Furthermore, a study by Canto and colleagues confirmed the utility of clinical surveillance to detect pancreatic cancers early and improve patient outcomes. Out of 354 high-risk individuals, meaning those with a family history of PDAC or a pathogenic germline variant in a pancreatic cancer susceptibility gene, 48 (13.6%) had surgical resections, with nine (2.5%) being confirmed as PDAC. Importantly, the five-year survival rate for patients with PDAC was 60%. Moreover, a study by Abe and colleagues found that in 464 high-risk individuals, those with a pathogenic germline variant in a pancreatic cancer susceptibility gene, were more likely to progress to PDAC, compared to those without a pathogenic germline variant (HR = 2.85) [57].

Utilizing tumor histomorphology and a mutation spectrum to aid in the diagnosis of pancreatic cancers driven by *ATM* loss is an intriguing prospect, particularly for patients with a germline *ATM* variant of unknown significance or equivocal somatic data. In *ATM*-associated breast tumors, Renault and colleagues noted histological differences of *BRCA1*-associated tumors. In addition, a mutation spectrum associated with *BRCA1/BRCA2*-deficient cancers, including PDAC, was defined [46,58,59]. However, Hutchings and colleagues found diverse histologic subtypes in patients with PDAC and a pathogenic germline *ATM* variant, albeit with a statistically significant excess of colloid carcinomas [60]. Furthermore, a mutation spectrum specific to *ATM* loss has not yet been identified in PDAC [61]. Further, large-scale studies of pancreatic tumors from patients with pathogenic germline variants are needed to determine the utility such approaches.

The role of therapy targeted at the tumoral loss of *ATM* in the treatment of PDAC patients is unknown but is a subject of intense research due to the potential sensitivity of PDAC cells to radiation therapy, chemotherapy, and agents exploiting synthetic lethal strategies by targeting DNA repair [50]. A study by Ayers and colleagues assessed sensitivity to radiation therapy and selected chemotherapeutic agents in Panc2.5, MIA PaCa-2, and Panc8.13 cells, with *ATM* knocked down using shRNA, and demonstrated significant sensitivity to radiation therapy but not to the chemotherapeutics tested, including gemcitabine, topotecan, doxorubicin, olaparib, trametenib, cisplatin, mitomycin c, an *ATR* inhibitor (Ve821), and a DNA-Pkcs inhibitor (Nu7441) [62]. While the observed radiation sensitivity in PDAC cells confirms radiation sensitivity seen in *ATM*-deficient neoplastic and non-neoplastic cells, the results of chemosensitivity testing conflicts with evidence from some in vitro, in vivo, and clinical studies. 

Proteins downstream of *ATM*, chiefly *ATR* and *CHK1/2*, are also potential targets for therapy in patients with pancreatic cancer. *Chk1* inhibitors are known to sensitize pancreatic cancer cells to chemotherapy and radiotherapy [63,64]. Synthetic lethal interactions between *ATM* and *ATR* have also been observed, with *ATM*-deficient pancreatic cancer cell lines showing growth inhibition to *ATR* inhibitors [65]. These studies suggest that targeted therapies of downstream targets in *ATM*-deficient cancers may provide a promising approach for the treatment of patients with pancreatic cancer. 

Pathogenic germline *ATM* variants are one of the most frequently inherited variants associated with a high risk of PDAC identified in patients with PDAC and/or IPMN, irrespective of family history. As such, individuals with a pathogenic germline *ATM* variant are potential candidates for clinical surveillance to identify cancers early before they have spread, and surgical intervention may be curative. Furthermore, the somatic loss of *ATM* may point to therapeutic vulnerabilities that may in the future be exploited in the care of patients with PDAC.

## Figures and Tables

**Table 1:** Pathogenic germline *ATM* variants in patients with pancreatic ductal adenocarcinoma (PDAC).

Patients	Total Number of Patients	Number of Pathogenic Germline *ATM* Variants	Percentage (%)	Reference
**FPC**	166	4	2.4	[19]
	54	2	3.7	[22]
	638	21	3.4	[24]
	185	6	3.2	[8]
**Nonfamilial PDAC**	854	10	1.2	[3]
	149	3	2	[30]
	117	2	1.7	[8]
	350	4	1.3	[32]
**Unselected PDAC**	290	3	1.0	[21]
	96	4	4.2	[23]
	176	5	2.8	[29]
	3030	69	2.3	[25]
	1213	46	3.8	[26]
	298	10	3.3	[31]
	615	11	1.8	[33]
	3594	48	1.3	[34]
	289	4	1.4	[27]

FPC = patients with familial pancreatic cancer (two affected first-degree relatives in kindred), nonfamilial PDAC = patients with pancreatic adenocarcinoma and no family history of PDAC or family history that does not meet criteria for FPC, and unselected PDAC = patients with PDAC with or without a family history of PDAC.
